# Medial malleolar osteotomy in the treatment of osteochondral lesions of the Talus – incidence and impact on functional outcome: a retrospective analysis based on data from the German Cartilage Register (KnorpelRegister DGOU)

**DOI:** 10.1186/s12891-024-07541-8

**Published:** 2024-06-01

**Authors:** Lukas Klein, Gerrit Bode, Markus Wenning, Max Behrens, Hagen Schmal, Jan Kühle

**Affiliations:** 1 Department of Orthopedics and Trauma Surgery, Faculty of Medicine, Medical Center, University of Freiburg, Hugstetter Straße 55, 79106 Freiburg, Germany; 2 Praxisklinik 2000, Freiburg, Germany; 3 BDH-Klinik Waldkirch, Waldkirch, Germany; 4 Drescher Orthopädie, Freiburg, Germany; 5 Institute of Medical Biometry and Statistics, Faculty of Medicine and Medical Center, University of Freiburg, Freiburg, Germany

**Keywords:** Osteochondral lesions of the talus (OCLT), Medial malleolar osteotomy (MMO), Patient-reported outcome measures (PROMs)

## Abstract

**Background:**

There has long been discussion regarding the impact of medial malleolar osteotomies (MMO) as an adjunctive treatment for osteochondral lesions of the talus (OCLT). MMO may improve the visibility and accessibility of the talus, but they also pose a risk of periprocedural morbidity. There is a lack of research about the prevalence and consequences of MMO in the surgical treatment of OCLT.

**Methods:**

This study retrospectively evaluated data from the German Cartilage Register (KnorpelRegister DGOU) from its implementation in 2015 to December 2020. The impact of MMO on patient-reported outcome measures (PROMs) was investigated. Wherever possible, subgroups were built and matched using a propensity score which matched a group undergoing OCLT without MMO. Matching included age, sex, weight, localization of the OCLT, the international cartilage repair society (ICRS) grading, surgical procedure and preoperative symptoms using the Foot and Ankle Ability Measure (FAAM) and the Activities of Daily Living Subscale (ADL).

**Results:**

The prevalence of MMO in the operative treatment of OCLT was 15.9%. Most of the osteotomies were performed in OCL of the medial talar dome (76.8%) and in more serious lesions with an ICRS grade of III (29.1%) and IV (61.4%). More than half of the osteotomies (55.6%) were performed during revision surgery. A matched pair analysis of *n* = 44 patients who underwent AMIC^®^ via arthrotomy and MMO vs. arthrotomy alone showed no significant differences in patient-reported outcome measures (PROMs, i.e. FAAM-ADL, and FAOS) at 6,12 and 24 months.

**Conclusions:**

MMO are mostly used in the treatment of severe (≥ ICRS grade 3) OCL of the medial talar dome and in revision surgery. Functional and patient-reported outcome measures are not significantly affected by MMO compared to arthrotomy alone.

**Trial registration:**

The German Cartilage Register (KnorpelRegister DGOU) was initially registered at the German Clinical Trials Register (https://www.drks.de, register number DRKS00005617, Date of registration 03.01.2014) and was later expanded by the ankle module.

## Background

The surgical treatment of osteochondral lesions (OCL) of the talus (OCLT) follows a stage dependent approach [1, 2]. Independently of the chondroregenerative procedure, many surgeons perform a medial malleolar osteotomy (MMO) to address OCLT, especially of the medial talar dome [3, 4]. This is a technique which offers an improved visualization of the defect, facilitating maneuverability during cartilage repair more than arthrotomy alone [5]. However, there has long been discussion surrounding the locations of OCLT it should be performed for and whether it affects mid- and long-term outcomes [4, 6]. In-vitro studies have previously shown, that the majority of the talus can be reached with arthrotomy alone [6]. Muir et al. claim this area comprises 75% of the talus’ surface [7]. Respectively, there is only a residual space of 17% of the posterior medial talus which may require osteotomy. This assumption is also backed by investigations of antero- and posteromedial arthrotomy, demonstrating an area of 20% of the talus where MMO can be helpful for treating an OCL properly [6]. Clinical studies have demonstrated that OCL of the medial talus can be successfully treated without performing an osteotomy at all [1, 8].

Since an MMO may be associated with increased periprocedural morbidity due to the increased invasiveness of the procedure, it needs to be thoroughly considered. Implant removal as an additional surgical procedure might also be required. While some authors describe no short- or mid-term morbidity, other studies suggest this rate to be as high as 30% [4, 9, 10]. The current literature does not provide sufficient evidence for a conclusion to be drawn. Furthermore, the rate of postoperative complications after MMO, such as malunion and displacement, remains unclear. This is in part due to the fact that multi-center and long-term investigations with a great number of patients are difficult to realize. Patient data from large registries has been shown to be of sufficient quality to overcome this difficulty.

This study therefore aims to calculate the incidence and evaluate the impact of MMO on the mid-term outcomes of OCLT, by analyzing data from the German Cartilage Register (KnorpelRegister DGOU).

## Methods

### Ethical statement

The study was performed in accordance with the ethical standards laid down in the 1964 Declaration of Helsinki and its later amendments. It was approved by the local institutional review board, the Ethics Committee of the University of Freiburg (Ethik-Kommission der Albert-Ludwigs Universität Freiburg), Freiburg, Germany (ETKFR #520/14, November 2014).

### Data source

We retrospectively analyzed data from the German Cartilage Register (KnorpelRegister DGOU) which is financially supported by the German Arthritis Foundation (Deutsche Arthrose Hilfe e.V.) and the Oskar and Helene Heim Foundation (Stiftung Oskar-Helene-Heim). Patients included in this registry report their preoperative symptoms and their postoperative recovery via established and standardized online questionnaires that are sent to them at regular intervals [8, 11]. Data is then collected and administered by the center for clinical studies Freiburg (ZKS Freiburg). All the patients included in the ankle module of the register from 2015 up to December 2020 were screened for eligibility. In total, 52 centers included patients to the ankle module. Up to the time of analysis, *n* = 904 patients had been included into the ankle module of the database. Their demographic details, the etiology of OCL, symptoms, defect classification, surgical treatment and patient-reported outcome measures (PROMs) were collected.

### Data selection

**Fig. 1:**
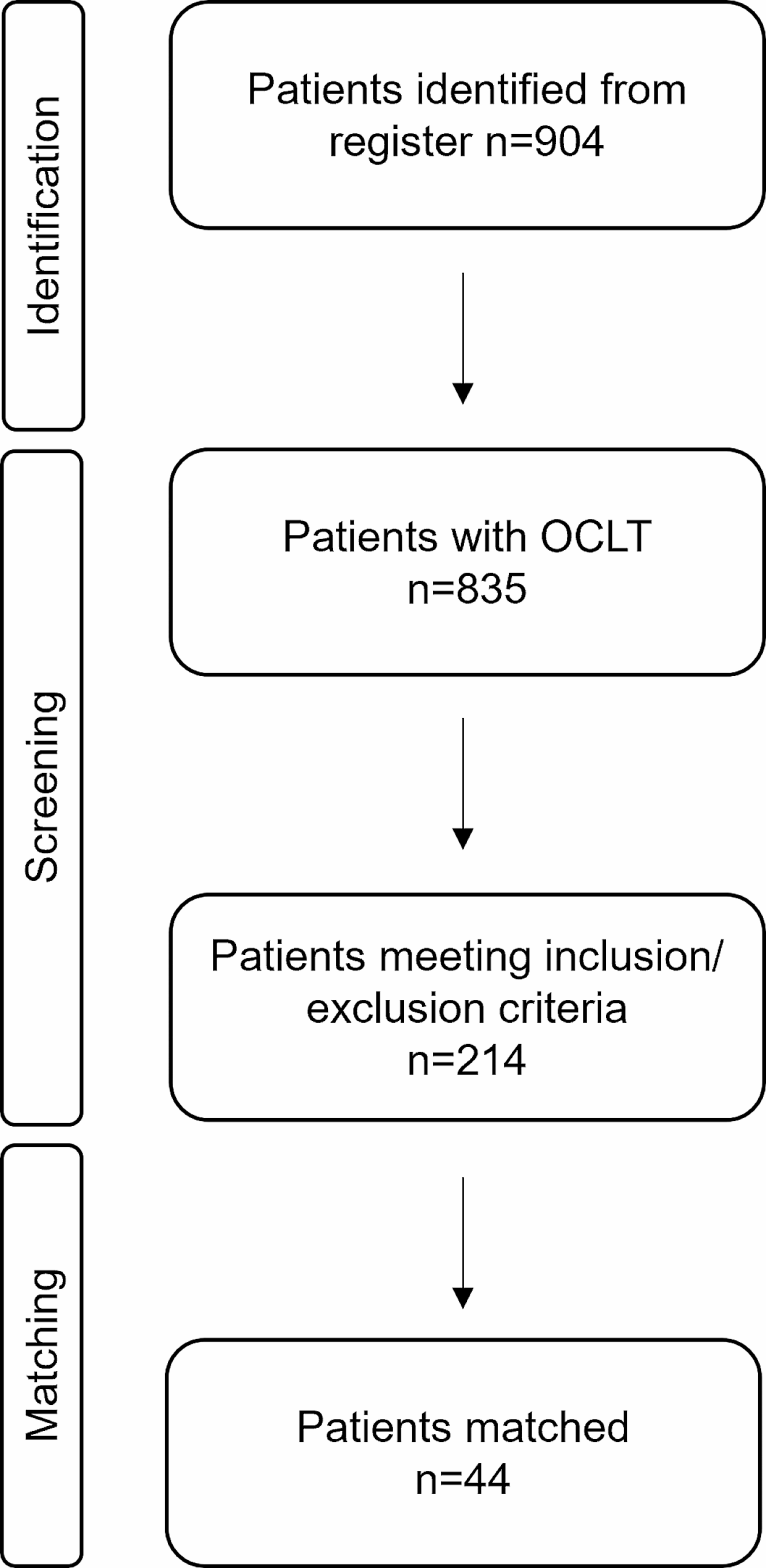
Screening for eligibility and matching

All patients who underwent an MMO in the treatment of their OCLT were first investigated for prior treatment, the location and surgical intervention. To investigate the influence of an MMO on the postoperative outcome, patients with isolated OCL who underwent their first surgical intervention via arthrotomy alone or combined with a MMO were identified (Fig. 1).

Inclusion criteria were:

- Singular osteochondral lesion of the talus (OCLT).
- Surgical approach via arthrotomy alone or combined with an MMO.

Exclusion criteria were:

- Previous surgical treatment at the affected ankle/ lesion.
- Missing data (i.e. operative procedure, matching criteria, outcome).

Groups were then matched concerning age, sex, weight, localization of the OCL, the International Cartilage Repair Society (ICRS) grading, surgical procedure and preoperative symptoms using the Foot and Ankle Ability Measure (FAAM) and Activities of Daily Living Subscale (ADL).

### Classification

OCL were classified according to their location on the talar surface and the grade of the lesion using the International Cartilage Repair Society (ICRS) grading [12]. The cartilage was classified as normal (ICRS grade 0), nearly normal in superficial lesions (ICRS grade I), abnormal in lesions extending down to 50% of the cartilage depth (ICRS grade II), severely abnormal in lesions extending down above 50% of the cartilage depth (ICRS grade III) and severely abnormal in lesions with subchondral bone involvement (ICRS grade IV).

### Outcome

The postoperative outcome was evaluated by the Foot and Ankle Ability Measure, Activities of Daily Living (FAAM-ADL) Subscale and the Foot and Ankle Outcome Score (FAOS) at 6, 12 and 24 months. The scores were translated into German and validated by van Bergen and Nauck [13, 14]. The FAAM ADL contains 21 questions about patient symptoms for the affected ankle during activities such as standing, walking and working [14]. Every activity is rated by the patient from 0 (not possible to perform task) to 4 (no symptoms at all). The best score in asymptomatic patients is 84 and the worst is 0 points. The FAOS contains 42 questions about more specific symptoms like pain, stiffness of the ankle, difficulties during sport and quality of life [13]. Patients score each question on a Likert scale from 0 (extreme symptoms) to 4 (no symptoms) and results are then transformed on a scale from 0 (worst score) to 100 (best score).

### Statistical analysis

The statistics application R version 4.0.4 (R Foundation for Statistical Computing, Vienna, Austria) was used to perform a balanced, 1:1 matched pair analysis using propensity score matching. We paired with regards to age, sex, weight, localization of the osteochondral lesion, ICRS grade, surgical procedure and preoperative symptoms using the FAAM-ADL. The differences between the groups were tested through questionnaires using the Wilcoxon Rank Sum test, since the assumption of equal variance for the t-test seems to have been violated.

## Results

### Patient characteristics

A total of *n* = 835 patients were operated on for the treatment of an OCLT between 2015 and 2020, of whom 15.9% (*n* = 133) underwent an MMO. 57.1% (*n* = 76) were male and 42.9% (*n* = 57) were female (Table 1). The mean age was 33.8 years old (minimum 18, maximum 66), the mean weight was 83.43 kg and the mean height 173.28 cm. 44.4% (*n* = 59) of patients had no prior surgery at the affected ankle, 39.1% (*n* = 52) had one, 14.3% (*n* = 19) two and 2.3% (*n* = 3) three prior interventions. In 96.2% (*n* = 128) a singular lesion, in 2.3% (*n* = 3) two lesions and in 1.5% (*n* = 2) of cases three lesions were treated.

**Table 1:** Characteristics of patients in the ankle module who underwent an MMO in the treatment of an OCLT. y, years; kg, kilogram; cm, centimeter; MMO, medial malleolar osteotomy; OCLT, osteochondral lesion of the talus

Patient characteristics	
	**Patients with OCLT and MMO** ***n*** **=** **133(100**%**)**
Age in y, mean (SD)	33.8 (12.6)
Weight in kg, mean (SD)	83.4 (19.5)
Height in cm, mean (SD)	173.3 (13.6)
Sex	
male	76 (57.1%)
female	57 (42.9%)
Prior surgeries	
0	59 (44.4%)
1	52 (39.1%)
2	19 (14.3%)
3	3 (2.3%)
Treated lesions	
1	128 (96.2%)
2	3 (2.3%)
3	2 (1.5%)

### Lesion profile

**Fig. 2:**
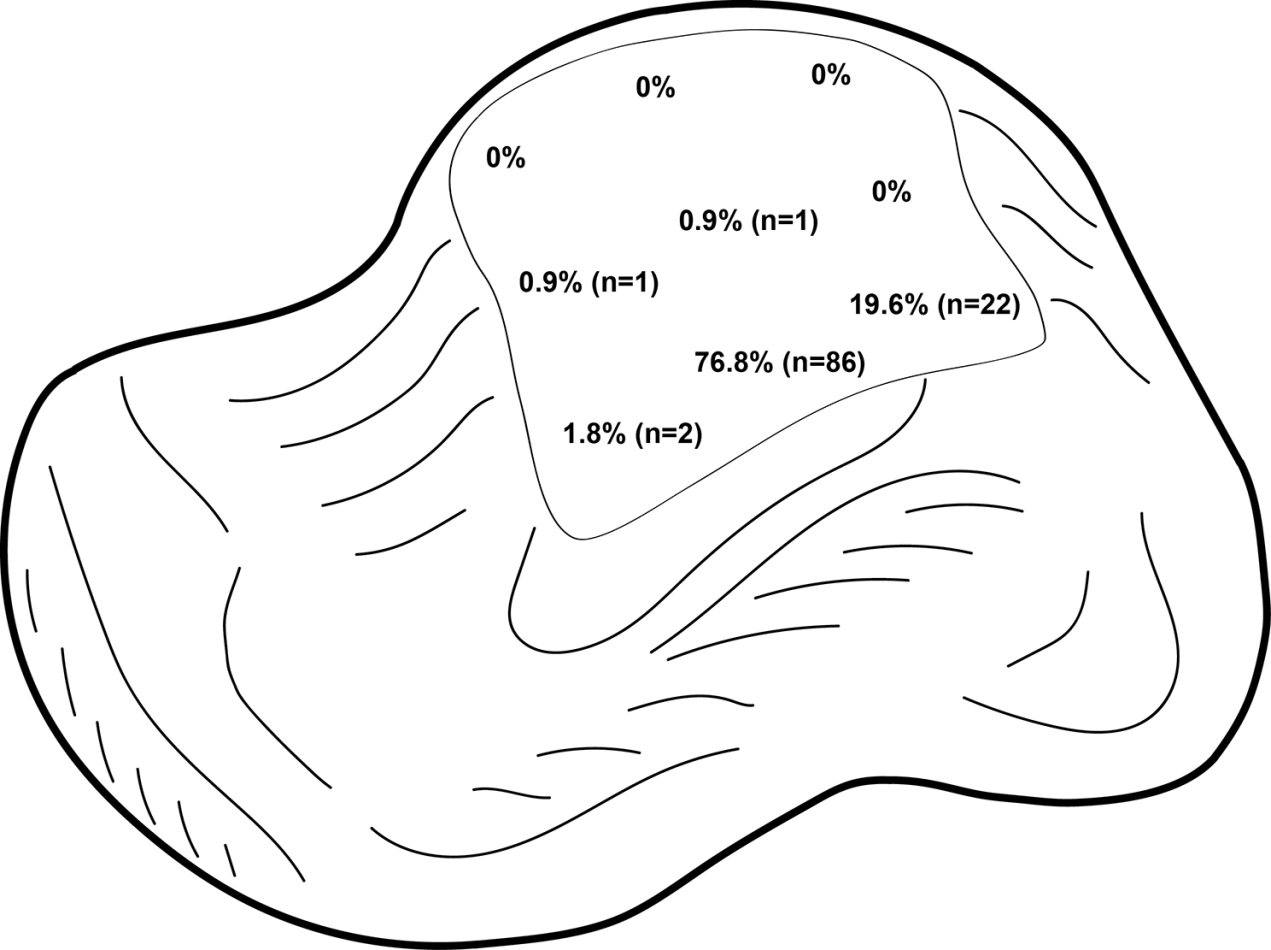
Location of singular OCLT which underwent an MMO. Nearly all are located on the medial part, with 98.2% (*n* = 110) and few on the central part (1.8%, *n* = 2). The medial talar dome was the most common localization with 76.8% (*n* = 86), followed by the medial posterior part with 19.6% (*n* = 22) of the lesions. All together there were 112 lesions, with missing information of *n* = 16

A total of *n* = 128 patients underwent surgery for a singular OCLT combined with a MMO. The exact localization of the OCLT was documented in 112 cases. Most of the lesions were located medially (98.2%, *n* = 110), with few on the central part (1.8%, *n* = 2). The medial talar dome was the most common localization with 76.8% (*n* = 86), followed by the medial posterior part with 19.6% (*n* = 22) of the lesions (Fig. 2).

In cases with singular lesions (*n* = 128) the ICRS grade was IV in 61.4% (*n* = 78), III in 29.1% (*n* = 37), II in 5.5% (*n* = 7) and I in 3.9% (*n* = 5) of cases. In one case, documentation was inconsistent and it was thus excluded from the analysis.

### Influence of the MMO on the postoperative outcome in AMIC^®^

A total of *n* = 44 patients with singular OCLT and without prior surgical intervention at the affected ankle were included in this analysis. Of those, *n* = 22 (50%) underwent arthrotomy alone and another *n* = 22 (50%) underwent arthrotomy combined with an MMO. The mean age was 31.7 in the arthrotomy and 31.6 in the osteotomy group. 59.1% (*n* = 13) of patients were male and 40.9% (*n* = 9) were female in each group (Table 2). The osteochondral lesions treated were all ICRS grades 3 and 4. All the patients in both groups were treated with an autologous matrix-induced chondrogenesis (AMIC^®^).

**Table 2:** Patient characteristics after matching. y, years; kg, kilogram; n, number; SD, standard deviation; ICRS, international cartilage research society; MMO, medial malleolar osteotomy, FAAM-ADL, Foot and Ankle Ability Measure, Activities of Daily Living Subscale

Patient characteristics after matching		
	**Arthrotomy***n* **=** **22**	**MMO***n* **=** **22**
Age in y (mean SD)	31.68 (10.08)	31.64 (11.15)
Sex n (%)		
female	9 (40.9%)	9 (40.9%)
male	13 (59.1%)	13 (59.1%)
Weight in kg, mean (SD)	83.55 (20.61)	83.86 (25.45)
ICRS Grade (%)		
1	0	0
2	0	0
3	6 (27.3%)	6 (27.3%)
4	16 (72.7%)	16 (72.7%)
Preoperative FAAM-ADL, mean (SD)	59.56 (21.96)	56.59 (26.09)

Comparisons of FAAM and FAOS were performed at 6,12 and 24 months, postoperatively. Both groups showed an improvement in the FAAM-ADL and FAOS over time. There was no significant difference in PROMs between the arthrotomy and the MMO groups (Tables 3 and 4).

**Table 3:** Foot and Ankle Ability Measure (FAAM), Activities of Daily Living Subscale (ADL) scores for the arthrotomy and medial malleolar osteotomy (MMO) subgroup. Presented are the median and the interquartile range (IQR)

	Arthrotomy (*n* = 22)	MMO (*n* = 22)	*p*
FAAM-ADL 6 months	73.21 (64.58, 86.61)	72.02 (50.60, 83.93)	0.511
FAAM-ADL 12 months	89.97 (76.49, 94.35)	80.00 (58.93, 94.05)	0.417
FAAM-ADL 24 months	94.05 (94.05, 97.62)	86.90 (79.76, 95.24)	0.172

**Table 4:** Foot and Ankle Outcome Score (FAOS) for the arthrotomy and medial malleolar osteotomy (MMO) subgroup. Presented are the median and the interquartile range (IQR)

	Arthrotomy *n* = 22	MMO *n* = 22	*p*
FAOS 6 months	86.76 (73.53, 94.12)	79.41 (64.71, 95.59)	0.423
FAOS 12 months	95.59 (85.29, 98.53)	87.50 (68.75, 97.43)	0.330
FAOS 24 months	95.59 (93.01, 99.26)	93.38 (91.54, 97.79)	0.476

## Discussion

### Patient characteristics

There were slightly more male than female patients in this cohort, and the mean patient age was 33.8. Previous studies show differing results, with some stating that women show higher incidences and others reporting men as having higher incidences of OCLT [15–17]. For example Orr et al. report that females in the united states military were more likely to develop OCLT [15]. Kim et al. also show that patients suffering from chronic ankle instability and OCLT are more often female [16]. It is possible that most of the patients in this cohort had a different etiology (i.e. traumatic) of their OCLT and we therefore found more male patients. Orr et al. also found that patient age is a significant risk factor in developing OCLT [15]. The observed mean age of 33.8 years in this cohort is comparatively old.

### Lesion profile and MMO

In this study, 75.9% of OCLT treated with an MMO were found on the medial talar shoulder. As summarized by Bruns et al. this is the most common location [17]. Raikin et al. developed an anatomical grid scheme and found that only 3.7% of OCLT are found on the central talar surface [18]. In the examined cohort this percentage was 1.8% in the population of all OCLT who underwent MMO and 0% after matching. Evidently, MMO is mostly performed for OCLT of the medial talar shoulder, rarely for the central and never for lesions of the lateral talar surface.

Meisterhans et al. stated that MMOs are foremost used in revision surgery or larger, cystic OCLT of the talar dome [3]. The findings in the present work support this claim, as 55.6% of MMOs were performed in patients who had prior surgery at the affected ankle. The majority of lesions in these patients were singular, and only a small percentage (3.8%) of patients had multiple lesions.

### Impact of the medial malleolar osteotomy on postoperative outcome

Our findings demonstrate continuous improvement of PROMs up to 24 months following AMIC^®^ procedure in both the MMO and arthrotomy group. Gottschalk et al. and Migliorini et al. previously demonstrated that PROMS improve significantly following AMIC^®^ and that it is superior to other procedures, such as microfracturing [8, 19]. However, most studies like the one from Kubosch et al. have focused on postoperative outcome in general and did not investigate the influence of the MMO [20]. Due to strict inclusion and exclusion criteria, the *n* = 22 patients in the MMO group form a very homogenous group and allowed for an elaborate matching, including preoperative symptoms, age and weight as the three most influential factors in this regard [16, 17]. Notably, this study found no influence of additional MMO on the postoperative outcome. Gottschalk et al. also compared the outcomes of 15 patients who underwent a MMO with 30 controls, in whom the operative approach was arthrotomy alone in the treatment of OCLs with MBS + I/III collagen scaffold (matrix-augmented bone marrow stimulation) [8]. Their follow-up was limited to 12 months and there was no significant difference between the two groups either.

There has been discourse about the true extent of postoperative complications following MMO, and Bull et al. report a malunion rate of up to 30% with 38.3% showing incongruence at postoperative radiographs [9]. This would suggest that a MMO negatively impacts the postoperative outcome. In the present study the true extent of complications following MMO was not investigated. But PROMs do not differ when compared to arthrotomy alone which indicates that regular postoperative complications that negatively impact perceived patient outcome seem unlikely. This claim is supported by Leumann et al. and C. Götze et al. who found no intraoperative complications as well as no malunions following MMO [4, 9]. Therefore it can be concluded that MMO does not negatively affect the postoperative outcome following AMIC^®^ when compared to arthrotomy alone.

## Limitations and strengths

The current study was designed as a retrospective study. Due to the strict inclusion criteria only 44 patients were included into the final analysis. This is a small sample compared to over nine hundred patients in the register. In addition to this, only few patients were operated at the same hospital or even by the same surgeon. This reduces the risk of bias introduced by the surgeon or the medical center. No correction with regard to the osteotomy technique was performed as it was not documented in the register.

## Conclusions

Surgical approaches with an MMO and arthrotomy alone in the treatment of OCLT lead to comparable patient-reported outcomes. We therefore argue that the use of an MMO can be advocated in cases where good exposure of the lesion is necessary to guarantee optimal surgical treatment.

## Data Availability

The datasets used and/or analyzed during the current study are available on reasonable request from Lukas Klein, Hugstetter Straße 55, 79106 Freiburg, Germany. E-Mail: lukas.klein@uniklinik-freiburg.de, or directly from the German Cartilage Register study center, e-Mail: info@knorpel-register.info.

## Abbreviations

ADL: Activities of Daily Living Subscale
FAAM: Foot and Ankle Ability Measure
FAOS: Foot and Ankle Outcome Score
MMO: Medial Malleolar osteotomy
ICRS: International Cartilage Repair Society
OCL: Osteochondral lesion
OCLT: Osteochondral lesion of the talus
PROMs: Patient-reported outcome measures
